# Environmental Factors Influencing Metal Concentrations in *Scomber colias* Along the Canary Islands

**DOI:** 10.1007/s12011-024-04250-0

**Published:** 2024-06-14

**Authors:** Enrique Lozano-Bilbao, Alba Jurado-Ruzafa, José María Lorenzo, José Antonio González, Arturo Hardisson, Dailos González-Weller, Soraya Paz, Carmen Rubio, Ángel José Gutiérrez

**Affiliations:** 1 Department of Obstetrics and Gynecology, Pediatrics, Preventive Medicine and Public Health, Toxicology, Legal and Forensic Medicine and Parasitology, Area of Toxicology, University of La Laguna, Campus de Ofra, San Cristóbal de La Laguna, Santa Cruz de Tenerife, 38071 Spain; 2 Grupo de Investigación en Ecología Marina Aplicada y Pesquerías (EMAP), Instituto de Investigación de Estudios Ambientales y Recursos Naturales (i-UNAT), Universidad de Las Palmas de Gran Canaria. Campus de Tafira, Las Palmas de Gran Canaria, Las Palmas, 35017 Spain; 3 Centro Oceanográfico de Canarias, Instituto Español de Oceanografía, Consejo Superior de Investigaciones Científicas (IEO, CSIC), Calle Farola del Mar n. 22, Santa Cruz de Tenerife, 38180 Spain; 4 Servicio Público Canario de Salud, Laboratorio Central. Santa Cruz de Tenerife, Santa Cruz de Tenerife, 38006 Spain

**Keywords:** Fish, Trace elements, ICP-OES, Anthropogenic

## Abstract

A total of 140 specimens of *Scomber colias* were collected from the Canary archipelago waters during the first semester of 2021, with 20 samples from each of the seven main islands. After analyzing the concentrations of metals (Al, Zn, Cd, Pb, Fe, and Cu) with ICP-OES, significant variations were observed among islands, with specimens from Tenerife and Gran Canaria containing higher levels of Al, Cd, and Pb, while those from Lanzarote and Fuerteventura had elevated levels of Zn, Fe, and Cu. These differences are probably related to greater anthropogenic activity around Tenerife and Gran Canaria coasts, leading to higher pollution levels, and the influence of Saharan dust and African upwelling on Lanzarote and Fuerteventura, enriching the waters with nutrients. Specific management strategies to mitigate marine pollution and continuous monitoring are crucial to safeguard marine ecosystems and to ensure food security.

## Introduction

Among others, marine pollution is known to be linked to population density in coastal areas and near urban zones, due to large quantities of wastewater often generated in areas with high population densities and, whether these waters are not properly treated, frequently, they flow directly into the ocean, carrying contaminants such as chemicals, excessive nutrients, and pathogenic microorganisms [1–5]. A higher population density also implies a greater generation of garbage and waste (including plastics, chemicals, heavy metals, and other non-biodegradable materials), which often are eventually dumped directly into the sea [2, 6, 7]. Likewise, as a result of illegal dumping, poorly managed waste, or leaching from landfills, other widespread used products containing heavy metals (such as paints, batteries, electronics, and pesticides) can end up and accumulate in the marine water [8–12]. All these residues and pollutants contaminate the water, harm marine life, and affect the quality of coastal ecosystems.

The Canary Islands face major challenges regarding marine pollution management, due to the great human pressure along the islands’ coastlines linked to tourism activities, which represents the major source of income in the archipelago [13–15]. On the other hand, noticeable differences in the concentration of some elements have been found in the recognized bioindicator species *Anemonia sulcata* in the archipelago, reflecting quick influence of several natural processes [16].

The Atlantic chub mackerel, *Scomber colias*, is a widely distributed medium pelagic fish found in coastal and oceanic waters in the Atlantic Ocean, including the Canary Islands archipelago [17]. While *S. colias* is not considered a specific bioindicator of pollution, it has been used in scientific studies to assess the presence and effects of contaminants in the marine environment [18–20].

Based on Atlantic chub mackerel collected along the longitudinal geographic range in the Canary archipelago, the main objective of the study is to investigate, for the first time, the existence of any pattern in the content of metals and trace elements in the species, as well as to explore the effect of unequal impact of anthropic pressures in the coastal marine ecosystems and natural processes among islands, to explain the potential variations.

## Materials and Methods

### Sample

**Fig. 1:**
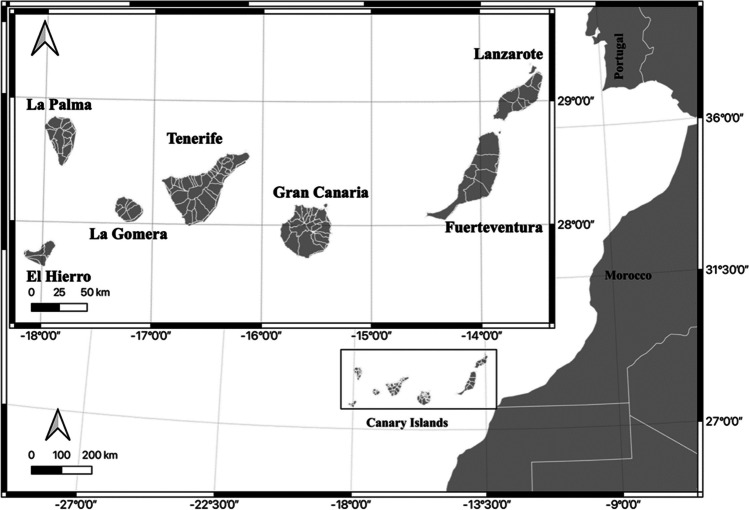
Map of the Canary Islands, indicating the islands where *Scomber colias* samples were collected

During the first semester of 2021, a total of 140 specimens of *Scomber colias* were collected along the Canary archipelago, 20 specimens from each of the seven main islands (W to E): El Hierro, La Palma, La Gomera, Tenerife, Gran Canaria, Fuerteventura, and Lanzarote (Fig. 1). The specimens were caught by fishermen in the eastern zone of the islands, to avoid the observed influence which volcanic activity (in the southern zone of El Hierro and the western zone of La Palma) has in concentrations of metals and traces in other marine organisms. The *S. colias* specimens were selected within a length range of 24 to 26 cm.

### Sample Preparation

Each specimen was processed by extracting 5 g of muscle tissue following the initial weight measurement. These tissue samples were then dried for 24 h in an oven set at 70 °C. Next, the dried samples underwent treatment in a muffle furnace at a temperature of 450 °C ± 25 °C until they turned into white ash. The resulting ashes were filtered and then adjusted to a final volume of 25 ml using a 1.5% HNO_3_ solution.

These samples were analyzed by inductively coupled plasma optical emission spectrometry (ICP-OES). Specifically, the ICAP 6300 model from Duo Thermo Scientific, Waltham, MA, USA, was used along with an attached auto sampler (CETAX model ASX-520). This analytical technique allowed the determination of the following elements: aluminum (Al), iron (Fe), cadmium (Cd), lead (Pb), copper (Cu), and zinc (Zn). A quality control solution was incorporated into the analysis after every ten samples to ensure the accuracy of the measurements. Moreover, the accuracy of the analytical procedure was evaluated by analyzing internationally recognized standard reference materials, specifically DORM-1 and DORM-5, provided by the National Research Council of Canada. The recovery rate achieved using these reference materials surpassed 97.5%. Blanks and standard reference materials were analyzed alongside the samples to assess the performance of the analytical method. Various validation parameters were examined, including specificity, precision (reproducibility), and accuracy (recovery). These parameters were evaluated by conducting ten measurements on each of the reference materials under reproducibility conditions. The verification process yielded the following outcomes: the method exhibited specificity, as no spectral interferences were observed for the metals under investigation; precision was confirmed for all metals, with a HORRATR value below 2; and accuracy was established by achieving a recovery rate exceeding 94% for all elements studied in the reference material. Consequently, the method used met the criteria for accuracy (recovery), precision (reproducibility), and specificity as specified in EC REGULATION No. 333/2007. The standards for the calibration curves were based on certified standard solutions. Specifically, for the metals (Al, Cd, Cu, Fe, Pb, and Zn), the SCP Science Multi-Element Std, SCP28AES certified standard was used, with a certified concentration of 100 mg/l for each of the metals. From these and, for each of the metals analyzed in the study, the different concentrations of the calibration standards were prepared for the elaboration of the calibration curves, all of them in sufficient quantity for 100 ml in 1.5% nitric acid. Instrumental conditions are the following: RF power, 1150 W; nebulizer and auxiliary gas flow, 0.5 l/min; coolant gas flow, 12.5 l/min; nebulizer gas pressure, 0.29 l/min; pump speed, 45 rpm.

### Statistical Analysis

A permutational multivariate analysis of variance (PERMANOVA) was conducted using Euclidean distances to explore potential variations in the content and relative composition of heavy metals and trace metals among the samples. The study followed a one-way design with the fixed factor of “island,” with seven levels of variation (El Hierro, La Palma, La Gomera, Tenerife, Gran Canaria, Fuerteventura, and Lanzarote). The analysis included the following variables: Al, Zn, Cd, Pb, Cu, and Zn. For the analysis, 9999 permutations of interchangeable units were used, and *post hoc* pairwise comparisons were conducted to assess differences between significant factors (*p*-value < 0.05). The statistical software packages PRIMER 7 and PERMANOVA þ v.1.0.1 were used to perform the analysis [21, 22].

## Results and Discussion

**Fig. 2:**
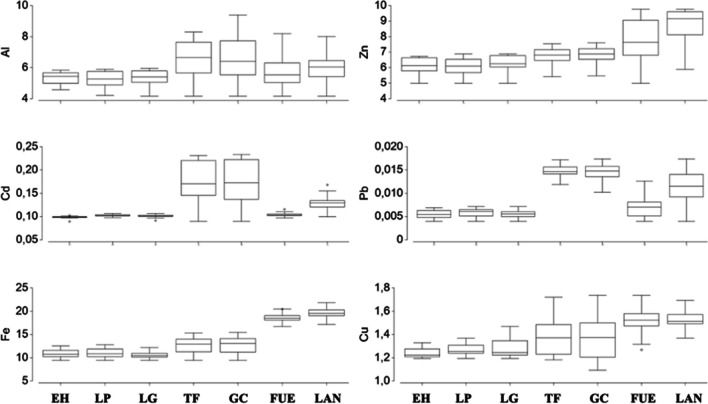
Box plot graphs for the concentrations of Al, Zn, Fe, Cu, Pb, and Cd (in mg/kg) obtained for *Scomber colias* collected from the seven islands of the study (from west to east). EH, El Hierro; LP, La Palma; LG, La Gomera; TF, Tenerife; GC, Gran Canaria; FUE, Fuerteventura; LAN, Lanzarote

**Fig. 3:**
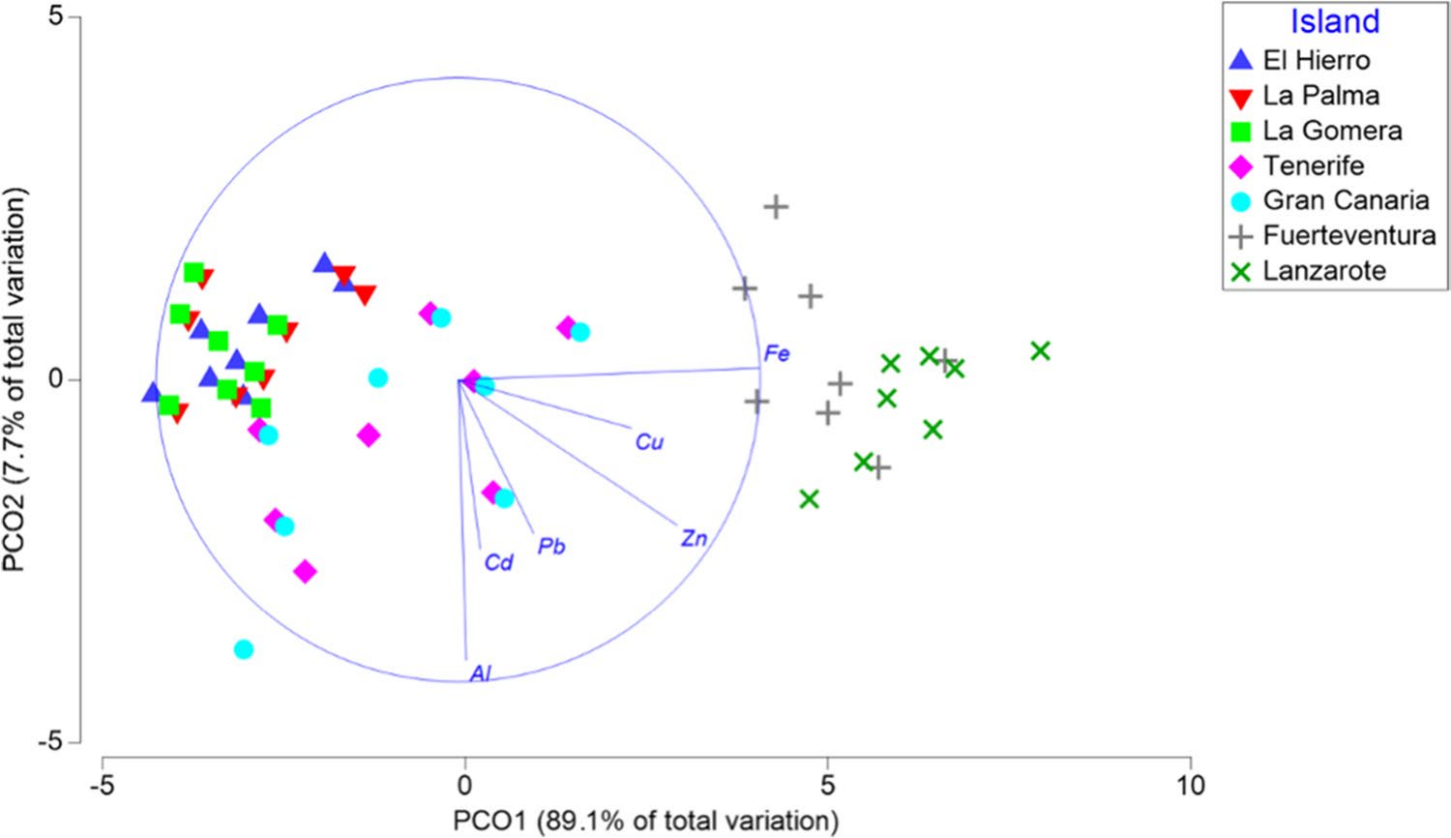
PCoA with the factor Islands 96.8% of total variation

Table 1 shows the mean concentrations of heavy metal and trace elements obtained in Atlantic chub mackerels by island in 2021. The specimens from Tenerife and Gran Canaria had the highest concentrations of Al, Cd, and Pb, while those from Lanzarote and Fuerteventura showed the highest concentrations of Zn, Fe, and Cu (Figs. 2 and 3).

**Table 1:** Mean values (± standard deviation) of the heavy metals and trace elements concentrations (in mg/kg) obtained for *Scomber colias* by island in 2021 (from W to E)

	Al	Zn	Cd	Pb	Fe	Cu
El Hierro	5.320 ± 0.437	6.095 ± 0.594	0.098 ± 0.004	0.006 ± 0.001	10.905 ± 1.020	1.242 ± 0.050
La Palma	5.237 ± 0.588	6.071 ± 0.596	0.103 ± 0.002	0.006 ± 0.001	11.068 ± 1.088	1.275 ± 0.054
La Gomera	5.319 ± 0.594	6.318 ± 0.444	0.101 ± 0.004	0.006 ± 0.001	10.513 ± 0.551	1.290 ± 0.090
Tenerife	6.665 ± 1.157	6.821 ± 0.480	0.179 ± 0.039	0.015 ± 0.001	12.847 ± 1.575	1.385 ± 0.184
Gran Canaria	6.733 ± 1.468	6.890 ± 0.485	0.179 ± 0.042	0.015 ± 0.002	12.850 ± 1.756	1.374 ± 0.213
Fuerteventura	5.582 ± 0.833	7.853 ± 1.356	0.105 ± 0.005	0.007 ± 0.002	18.640 ± 0.906	1.507 ± 0.115
Lanzarote	5.963 ± 0.634	8.854 ± 0.945	0.131 ± 0.017	0.012 ± 0.003	19.691 ± 1.135	1.526 ± 0.065

Comparisons between islands for each metal are presented in Table 2. For Al, the specimens from Gran Canaria presented the highest concentration, with no significant difference from the Al concentration obtained for the Atlantic chub mackerels from Tenerife. The lowest Al mean value was obtained for the specimens from La Palma, with no significant differences among the *S. colias* specimens collected from El Hierro, La Palma, and La Gomera, located in the west of the Canary archipelago. Similarly, no significant differences were found between the specimens from Lanzarote and Fuerteventura, the easternmost islands in the archipelago. One of the primary sources of marine aluminum contamination is the improper treatment of wastewater discharge, frequently containing high concentrations of aluminum due to the presence of cleaning products, detergents, industrial chemicals, and other materials containing this metal [23–25]. The highest values found for the specimens from Tenerife and Gran Canaria are probably due to these islands housing the highest population densities in the archipelago, experiencing greater anthropogenic pressure along their coasts compared to the others [13]. Despite aluminum being among the most abundant elements in the Earth’s crust, its presence in marine environments can produce adverse effects on organisms and ecosystems, and attention to its values and variations should be paid to avoid environmental issues.

**Table 2:**
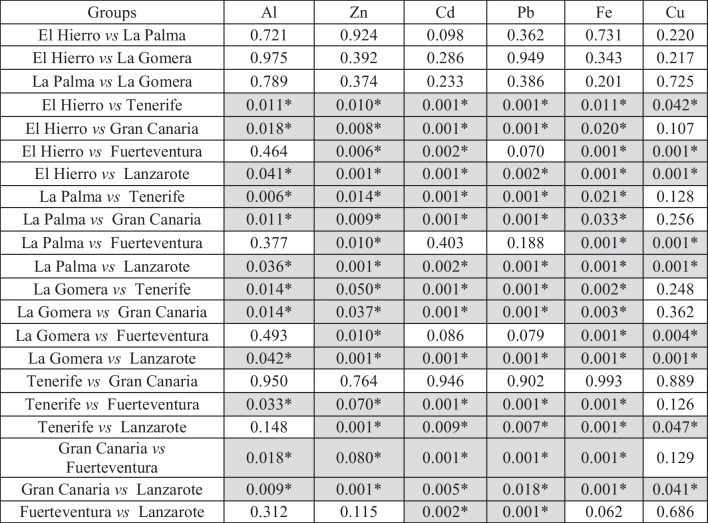
Results of pairwise tests examining the significant factor “island” obtained in a one-way ANOVA analyzing the metal content variation. *Significant difference (*p *< 0.05), grey shaded

Concerning Zn, there were no significant differences among the westernmost *S. colias* specimens (from El Hierro, La Palma, and La Gomera) nor between the specimens from Lanzarote and Fuerteventura and either between the specimens from the central islands (Tenerife and Gran Canaria). However, in this case, specimens from Lanzarote showed the highest concentration of Zn, while those from La Palma presented the lowest Zn concentration. Zinc in the ocean originates from various sources such as rock erosion, terrestrial sediments, volcanic activity, atmospheric emissions, biological activity, and rivers’ transportation of dissolved zinc and zinc particles. As an essential micronutrient for marine organisms, upwelling processes display a significant role in the Zn availability and function because it is transported from the deep ocean layers enriching surface waters. Likewise, desert dust contains a variety of minerals and compounds, including zinc. As the dust rises into the atmosphere and is carried by the wind, it can be deposited onto the ocean surface through dry deposition or be carried to the sea during precipitation events, known as wet deposition [26–32]. In this context, the easternmost islands of Fuerteventura and Lanzarote are the most influenced by the sandstorms originating in the Saharan Desert and by the major NW African upwelling, which could explain the greater Zn content found in the Atlantic chub mackerels analyzed from these islands, because 2020 and 2021 were years of high Saharan dust influence.

In the case of Cd, significant differences were found among the *S. colias* specimens from the western islands of El Hierro, La Palma, and La Gomera. Significant differences were found between the specimens from the easternmost islands, Lanzarote and Fuerteventura. Furthermore, no significant differences were observed among the specimens from Tenerife and Gran Canaria. As occurred for the Al, the specimens from Gran Canaria presented the highest Cd concentration at 0.179 ± 0.042 mg/kg, while the lowest Cd concentration (0.098 ± 0.004 mg/kg) was found in the specimens from El Hierro. Cadmium is a bioaccumulative (and potentially toxic) metal, meaning it tends to accumulate in the tissues of organisms along the biomagnification, having serious effects on aquatic ecosystems and posing a risk to human health. Cadmium is released into the marine environment as a result of human activity, being the agricultural runoff one of the main sources for the marine waters, due to Cd is found in some fertilizers and pesticides used in marine organisms such as fish and shellfish can accumulate cadmium in their tissues through their diet, so that, if humans consume seafood contaminated with cadmium, they may be exposed to health risks, as cadmium can be toxic to the nervous system, kidneys, and other organs [33–35]. In addition to the highest population densities, Tenerife and Gran Canaria have a larger number of crops, which become a potential source of Cd to the marine environments via runoff waters [36].

Again, no significant differences were found among the Pb contents in *S. colia*s from the western islands of El Hierro, La Palma, and La Gomera which presented the lowest Pb concentrations. Significant differences were found between the specimens from Lanzarote and Fuerteventura, the two easternmost islands in the archipelago, and between the specimens from Tenerife and Gran Canaria, with the highest Pb concentration. Marine contamination due to lead poses severe implications for both marine ecosystems and human health. Pb, being a toxic heavy metal, can infiltrate the marine environment through various human-related sources and activities. Significant contributors to marine lead pollution include lead-based pipes and paints used in port facilities and marine structures. As these pipes and paints corrode or wear down, they release lead particles into the water, elevating contamination levels. Lead poses toxicity to marine organisms, impacting their health, reproductive capabilities, and survival. Moreover, lead can bioaccumulate in aquatic organisms along the food chain, implying that organisms consuming lead-contaminated counterparts might accumulate even higher levels of this metal [37–41]. Due to the high population densities in Tenerife and Gran Canaria, resulting in greater anthropogenic pressure along their coastlines compared to other islands, as well as the presence of more and bigger ports, it is not surprising that specimens from these islands showed the highest concentrations of Pb. Lead particles can be suspended in the air, potentially originating from sources like tourist boats [42, 43].

As in the previous analyses, in the case of Fe content in *S. colias*, significant differences were found among the westernmost specimens (from El Hierro, La Palma, and La Gomera) between the specimens from Lanzarote and Fuerteventura and either between specimens from Tenerife and Gran Canaria. As found for the Zn, the samples from Lanzarote showed the highest concentration of Fe at 19.691 ± 1.135 mg/kg, although the lowest concentration of Fe at 10.513 ± 0.511 mg/kg was determined for La Gomera. Iron in the ocean can originate from various sources: erosion of continental rocks containing iron, dust and sediments carried by rivers, volcanic emissions, and human activities, such as the burning of fossil fuels. As occurs for Zn, deep waters and dust contribute to Fe water enrichment by upwelling and sandstorms processes [44–50]. As commented, Fuerteventura and Lanzarote are strongly influenced by Saharan dust and the NW African upwelling, which enrich the waters near these islands with nutrients, including iron. The Saharan dust is a meteorological phenomenon characterized by the presence of dust particles in suspension from the Sahara Desert, affecting the Canary Islands most frequently during the winter months, especially between December and February. During this period, atmospheric conditions, such as the easterly trade winds, are more conducive to transporting Saharan dust to the archipelago [51–53].

With respect to Cu contents, the same groups with no significant differences among the specimens of *S. colias* were found: a western group (El Hierro, La Palma, and La Gomera), an eastern group including the specimens from Lanzarote and Fuerteventura, and a central group with the specimens from Tenerife and Gran Canaria. However, the highest concentration of Cu was determined for the specimens from Lanzarote (1.526 ± 0.065 mg/kg), while specimens from El Hierro showed the lowest concentration of Cu at 1.242 ± 0.050 mg/kg. Copper in the ocean primarily originates from terrestrial sources, such as the erosion of copper-rich rocks and minerals (which may be transported to the oceans by rivers or sandstorms), as well as runoff from agricultural soils and urban areas where copper-containing products are used. Therefore, the upward flow of deep waters in the upwelling processes transports sedimented nutrients, including Cu, to the pelagic environment where the Atlantic chub mackerel develops. In addition, human activities such as mining and the release of industrial waste can introduce high amounts of copper into ocean waters [54–59]. The natural water enrichment in the easternmost islands of the archipelago is promoted both by the NW African upwelling and the Saharan dust, which seems to explain the higher concentrations of some of the analyzed elements, including copper.

### Comparison with Other Authors

Based on the literature available (Table 3), the highest concentration of Al in *S. colias* was found in the study by Lozano-Bilbao et al. [60], collected on the island of Tenerife in 2019, reaching 8.297 mg/kg. This high concentration could be attributed to the absence of trade winds in the Canary archipelago during the summer of 2017, resulting in significant warming of the ocean surface near the coast, which led to *Trichodesmium erythraeum* blooms, also known as *Trichodesmium* red tides. The occurrence of *T. erythraeum* blooms can depend on various factors such as water temperature, nutrient availability, and climatic conditions, which could have led to an accumulation of Al both in the plankton and in juveniles of *S. colias* hatched during winter 2018, which would have accumulated higher Al contents [61–63]. The highest concentrations of Zn (11.53 mg/kg) and Cu (6.02 mg/kg) in Atlantic chub mackerels were found in the study by [64] in Ghanaian waters, which could be due to limited regulations for pollution in the country, hence the elevated concentrations of metals and trace elements along the coasts. The highest concentrations of Cd and Pb were found in the present study and in the study by [20], in Tenerife where the high tourist density and the anthropic pressure could explain the high concentrations of these “anthropogenic nature” metals [2, 7, 65, 66]. Finally, the highest concentration of Fe was found in the present study in the specimens from the island of Lanzarote, reaching 19.691 mg/kg, probably explained by the proximity to the Sahara Desert and the NW African upwelling, which promote the coastal waters enrichment, with nutrients, including Fe.

**Table 3:** Content of metals and trace elements found in *Scomber colias* (mg/kg)

Study	Zone	Year	Al	Zn	Cd	Pb	Fe	Cu
Present study	El Hierro (Spain)	2022	5.320	6.095	0.098	0.006	10.905	1.242
	La Palma (Spain)		5.237	6.071	0.103	0.006	11.068	1.275
	La Gomera (Spain)		5.319	6.318	0.101	0.006	10.513	1.290
	Tenerife (Spain)		6.665	6.821	0.179	0.015	12.847	1.385
	Gran Canaria (Spain)		6.733	6.890	0.179	0.015	12.850	1.374
	Fuerteventura (Spain)		5.582	7.853	0.105	0.007	18.640	1.507
	Lanzarote (Spain)		5.963	8.854	0.131	0.012	19.691	1.526
[20]	Tenerife (Spain)	2016	7.4	7.906	0.06	0.138	15.437	1.37
[60]	Tenerife (Spain)	2017	8.297	7.732	0.051	0.127	15.108	1.281
[67]	Ghana	2017		2.2	0.01	0.069		2
[64]	Ghana	2020		11.53	0.079	0.059		6.02
[68]	Portugal	2019		6.8			10.5	
[69]	Portugal	2019		7.6	0.013	0.033		1.1
[70]	Azores (Portugal)	2010		21	0.027	0.028		1.6

The disparity in the concentrations of the metals Fe, Zn, and Cu in the species *S. colias* in the Canary Islands, with higher levels in the islands of Lanzarote and Fuerteventura compared to other islands, can be attributed to their proximity to the African continent and the influence of the Sahara. These islands, being closer to the African continent, are more exposed to atmospheric currents transporting particles and aerosols laden with metals from the Sahara. Local climatic conditions, such as the trade winds, may facilitate the deposition of these materials into the surrounding waters, resulting in higher concentrations of metals in marine organisms like *S. colias* [51, 53, 71]. On the other hand, the higher concentrations of Al, Cd, and Pb in the islands of Tenerife and Gran Canaria can be attributed to their higher population and tourism density, as well as increased industrial activity. Human activities, including urbanization, tourism, and industry, significantly contribute to environmental pollution and the release of heavy metals into the marine environment. Wastewater, industrial discharges, and vehicle emissions can serve as significant sources of metal pollution in these areas, leading to higher concentrations in marine organisms like *S. colias* [13, 15, 65, 72–74]. It is important to note that these differences in metal concentrations may have significant implications for the health of marine ecosystems and food security, as heavy metals can accumulate in the tissues of marine organisms and biomagnify along the food chain. Therefore, continuous monitoring of metal concentrations in marine organisms and appropriate measures to mitigate pollution are crucial to protecting the health of marine ecosystems and the human population that depends on them.

## Conclusions

The highest concentrations of the heavy metals and trace elements Al, Cd, and Pb were found in the specimens of *Scomber colias* from Tenerife and Gran Canaria. This is because these two islands have the highest population densities in the Canary archipelago, resulting in greater anthropogenic pressure along the coast compared to the other islands. On the other hand, the concentrations of Fe, Cu, and Zn were higher in the islands of Lanzarote and Fuerteventura. These islands are significantly influenced by Saharan dust and African upwelling, enriching the waters near the islands with nutrients.

The elevated concentrations of Al, Cd, and Pb in Tenerife and Gran Canaria can be attributed to factors such as higher population and tourism densities, as well as increased industrial activity in these central islands. Human activities, including urbanization, tourism, and industry, contribute significantly to environmental pollution, releasing heavy metals into the marine environment.

Overall, these disparities in metal concentrations underscore the importance of continuous monitoring and mitigation efforts to safeguard marine ecosystems and ensure food security. By understanding the sources and implications of metal contamination, policymakers and stakeholders can implement targeted strategies to mitigate pollution and protect both marine organisms and human populations reliant on them.

## Data Availability

No datasets were generated or analysed during the current study.
